# Multifunctional Prussian-Blue-Based Nanocomposite Hydrogel for Infected Wound Regeneration

**DOI:** 10.3390/gels11110895

**Published:** 2025-11-08

**Authors:** Pengchao Zhao, Zhishen Zhang, Dianhao Gong, Hongzhen Luo, Huiying Yu, Xin Li, Kun Lei, Chunshan Quan, Yun Xue, Lijun Guan

**Affiliations:** 1 College of Medical Technology and Engineering, Henan University of Science and Technology, Luoyang 471023, China; 2 Shanxi Key Laboratory of Yuncheng Salt Lake Ecological Protection and Resource Utilization, Yuncheng University, Yuncheng 044000, China; 3 Department of Biology, Xinzhou Normal University, Xinzhou 034000, China; 4 State Key Laboratory of Polymer Materials Engineering, Sichuan University, Chengdu 610065, China; 5 Department of Life Science, Dalian Nationalities University, Dalian 116600, China; 6 College of Animal Science and Technology, Henan University of Science and Technology, Luoyang 471023, China

**Keywords:** Prussian blue nanoparticle, Schiff-base hydrogel, photothermal ability, antibacterial property, wound healing

## Abstract

The wound healing (WH) process is often severely hindered by bacterial infections and prolonged inflammatory responses. To address this problem, we developed a novel injectable nanocomposite DPB-ODQ hydrogel, which comprises polydopamine-modified Prussian blue nanoparticles (PB@PDA, also called DPB) and an oxidized dextran/quaternized chitosan (QCS)-based Schiff-base network. This hydrogel possesses a highly interconnected porous structure, an excellent swelling rate (730%), rapid gelling speed (45 s), a high mass retention rate over a three-day period (73.20%), and exceptional self-healing properties. Based on the presence of PDA and the Schiff base, it also exhibited good adhesive strength (13.5 kPa). In addition, under near-infrared irradiation at 1.0 W/cm^2^, temperatures increased by more than 35 °C within 5 min, indicating excellent photothermal (PT) performance. The PT performance of DPB, synergized with the inherent antibacterial properties of QCS, endowed it with a bactericidal rate exceeding 96% against both *Staphylococcus aureus* and *Escherichia coli*. In vitro cell experiments have shown that it significantly promoted fibroblast proliferation and migration. In experiments involving mice infected with *S. aureus*, DPB-ODQ demonstrated an impressive WH rate of 92.82%, greatly promoting collagen deposition.

## 1. Introduction

As the largest organ of the human body, the skin constitutes the first line of defense against external physical, chemical, and biological threats [1]. Its intricate multi-layered structure—comprising the epidermis, dermis, and subcutaneous tissues—not only fulfills essential physiological functions such as protection, sensation, thermoregulation, and secretion but also performs complex repair procedures for injuries. When the integrity of the skin is compromised due to trauma, surgical procedures, burns, or chronic conditions such as diabetes mellitus, prompt and effective wound healing (WH) becomes critical for restoring barrier function, thwarting infection risks, and maintaining homeostasis. The classical WH process is a highly coordinated and dynamically continuous biological event, and it is typically delineated into four interrelated phases: hemostasis, inflammation, proliferation, and remodeling [2].

Wounds provide an entry point for environmental microorganisms. Bacterial infections are among the most prevalent and detrimental complications associated with WH; they significantly delay recovery while increasing patient suffering and healthcare burdens. They also result in severe outcomes, such as local tissue necrosis, systemic sepsis, or even death [3]. The underlying mechanism involves the colonization and proliferation of pathogens at the wound site, along with their release of toxins and enzymes, which damage host tissues. Furthermore, bacteria and their metabolic byproducts, such as lipopolysaccharides and exotoxins, can activate and sustain the host’s inflammatory response, resulting in the production of excessive reactive oxygen species (ROS) and proteases, which may inflict collateral damage on healthy tissues while clearing pathogens. It also disrupts neovascularization and hinders re-epithelialization, in addition to granulation tissue formation [4]. Therefore, the development of novel anti-infection strategies aimed at targeting biofilms and modulating excessive inflammation is urgently needed [5]. In this context, hydrogels have emerged as a prominent and attractive material choice for modern wound dressings (WDs) [6]. Their three-dimensional (3-D) porous architecture ensures adequate oxygenation and water vapor permeability, which are essential for wound metabolism while simultaneously preventing excessive moisture accumulation that could lead to maceration [7,8].

Chitosan (CS), as a natural cationic polysaccharide (PS), has been extensively utilized as an ideal raw material for WD due to its remarkable hemostatic properties and ability to promote cell migration. Nevertheless, the limited solubility of CS in water restricts its broader application. Quaternized chitosan (QCS), as a derivative of CS, not only enhances water solubility but also exhibits superior biocompatibility and stronger antibacterial efficacy [9,10]. Dextrin (Dex) is a neutral natural PS, and it has the ability to promote hemostasis, accelerate healing processes, and inhibit bacterial growth. The adjacent hydroxyl (–OH) groups within the Dex molecule can be oxidized to form aldehyde (–CHO) groups that readily cross-link with amino (–NH_2_)-containing substances through Schiff-base reactions.

Prussian blue (PB) is an FDA-approved clinical agent for the treatment of radioactive thallium and cesium poisoning [11,12,13]. PB nanoparticles (PB NPs) and their degradation products, such as ferrous ions, exhibit angiogenic properties that are essential for delivering oxygen and nutrients to wound tissue, clearing metabolic waste, supporting granulation tissue formation, and facilitating wound closure. They also convert light energy into heat under near-infrared (NIR) irradiation, generating localized high temperatures that rapidly eliminate various bacteria present in wounds—including resistant strains—thereby effectively controlling infections and promoting healing [14,15,16]. The introduction of a polydopamine (PDA) coating not only improves the dispersion stability of PB NPs but also imparts antioxidant characteristics through phenolic –OH and –NH_2_ groups [17]. Incorporating either PB or PB@PDA (DPB) NPs into hydrogel networks can yield smart dressings that exhibit mechanical adaptability and sustained antibacterial effects via controlled NP release [18,19]. Furthermore, they promote angiogenesis, facilitate collagen deposition, and accelerate WH [20,21].

Numerous studies have focused on wound dressings that utilize QCS, PB, and PDA, with notable contributions made by authors such as Hou et al. [22], who incorporated PDA into QCS/oxidized Dex (ODex) hydrogels to enhance their adhesion and antibacterial properties. Yu et al. [23] combined PB nano-hybrid hydrogels with specific far-infrared graphene devices to promote WH through thermal effects. However, they often fail to effectively address multifaceted challenges in the micro-environment of infected wounds. In this study, we presented an innovative multifunctional PB-based nanocomposite hydrogel (DPB-ODQ) that uniquely integrates multifunctional DPB NPs into a dynamic covalent network formed by QCS and ODex. This novel formulation combines the inherent adhesion and biocompatibility of QCS with the photothermal (PT) capabilities of DPB, thereby achieving high adhesion along with efficient PT antibacterial activity and tissue regeneration promotion within a single coordinated platform. We anticipate that this integrated treatment strategy will provide a superior solution for tackling the complex pathological processes associated with WH in infections.

## 2. Results and Discussion

### 2.1. Preparation and Structural Characterization of DPB-ODQ Hydrogels

**Figure 1:**
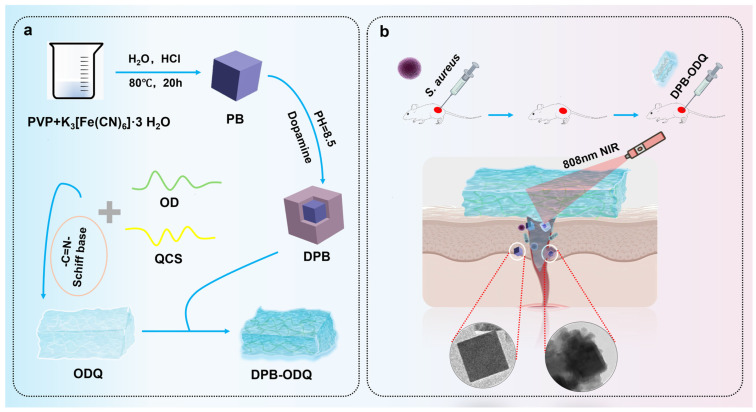
Schematic illustration of the preparation and application of hydrogels: (**a**) Schematic representation of hydrogel synthesis. (**b**) Application of hydrogel in wound care.

In this study, QCS was incorporated into hydrogels to enhance their adhesion properties, biocompatibility, and swelling characteristics [24]. It is likely that QCS alone exhibits limited antibacterial efficacy; therefore, DPB was introduced into these hydrogels as well. The incorporation of physical PT effects allows for precise and effective antibacterial outcomes, as thermal energy generated by NIR radiation from DPB can disrupt bacterial DNA and effectively kill bacteria [25]. Consequently, it is anticipated that DPB-ODQ hydrogels will introduce synergistic antibacterial effects through both QCS and DPB NPs. The overall strategy for synthesizing the DPB-ODQ hydrogel and its application in treating mouse wounds is depicted in Figure 1. The –CHO groups on the ODex chains react with the –NH_2_ groups of QCS through Schiff-base reactions, introducing imine bonds and a covalent cross-linked network that constitutes the fundamental framework of DPB-ODQ. Among them, the oxidation degree for ODex was determined as 55% (Figure S1), while that for QCS was 40%. The molar ratio of the –CHO to –NH_2_ groups utilized for cross-linking is 1.14:1. Subsequently, PDA-coated PB NPs are firmly integrated through multiple non-covalent interactions, including robust hydrogen bonding and metal coordination with catechol groups from the PDA layer and the –NH_2_ and –CHO groups within the polymer matrix. Concurrently, negative charges present on NP surfaces engage in electrostatic interactions with positive charges from QCS. Collectively, these non-covalent forces culminate in a stable 3-D cross-linked structure.

**Figure 2:**
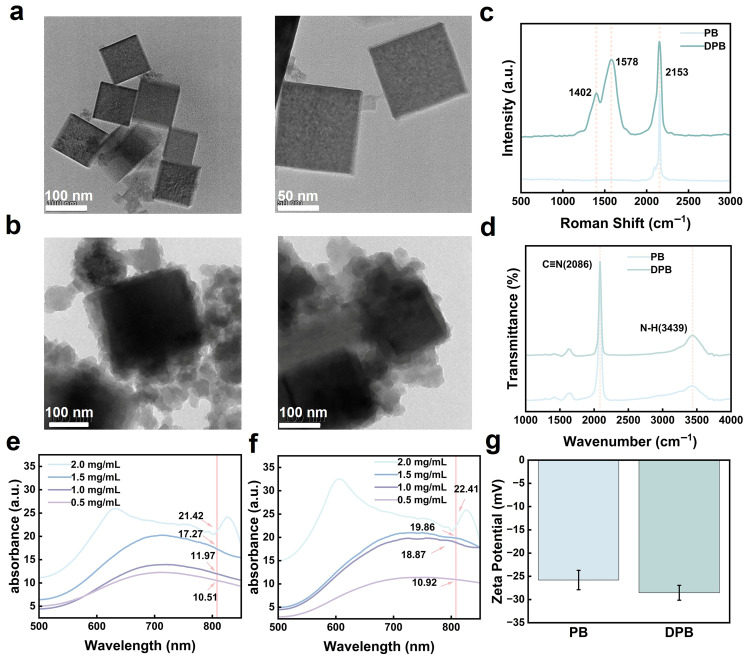
Structural characterization of PB and DPB NPs: (**a**) TEM images of PB. (**b**) TEM images of DPB. (**c**) Raman spectra of PB and DPB. (**d**) FTIR spectra of PB and DPB. (**e**) Absorbance of PB. (**f**) Absorbance of DPB. (**g**) Zeta potentials of PB and DPB (*n* = 3).

TEM images revealed that both PB and DPB NPs exhibited uniform morphology (Figure 2a,b), among which the average size of DPB was estimated to be around 115 nm (Figure S2). For bare PB, a Raman peak corresponding to the vibrational mode of –CN was observed at 2153 cm^−1^ (Figure 2c). In contrast, for DPB, two peaks at 1402 cm^−1^ and 1578 cm^−1^ were attributed to stretching and deformation modes within the aromatic ring, providing strong evidence for the presence of PDA. Furthermore, FTIR spectrum measurements indicated that bare PB displayed a characteristic peak at 2086 cm^−1^, which is associated with –CN stretching vibrations involving Fe^2+^–Fe^3+^, while a broad and intense band appeared around 3439 cm^−1^ for DPB, corresponding to N–H stretching vibrations originating from PDA (Figure 2d) [26]. The absorbance test showed that the OD values of different concentrations of DPB in the NIR region were higher than those of PB, indicating that DPB possesses superior absorption capacities compared to PB (Figure 2e,f). Due to the negative charge associated with the PDA layer, the potential of PB decreased upon the application of the PDA coating, changing from −25 mV to −28 mV; this directly reflected the orderly modification process experienced by PB (Figure 2g).

**Figure 3:**
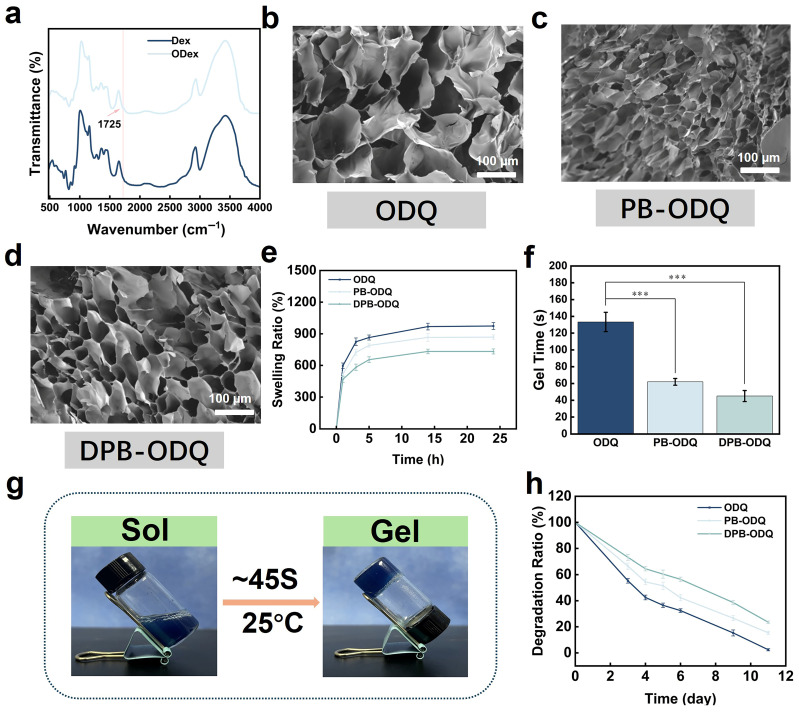
Structural characterization of hydrogels and their GT, swelling and degradation tests: (**a**) FTIR spectra for DEX and Odex: (**b**–**d**) SEM images of ODQ, PB-ODQ and DPB-ODQ, respectively. (**e**) Swelling behavior (*n* = 3). (**f**) Gelation time measurement (*** *p* < 0.001, *n* = 3). (**g**) Sol–gel transition diagram. (**h**) Degradation assessment (*n* = 3).

A new weak absorption peak at 1725 cm^−1^ was observed, which can be ascribed to carbonyl (–C=O) groups without any discernible –CHO signals, suggesting that the –CHO groups within ODex may form hemiacetals with adjacent –OH groups (Figure 3a). SEM images revealed highly interconnected porous structures in hydrogels (Figure 3b–d) [22]. While both PB and DPB contribute to a reduction in pore sizes, it appears that PB enhances cross-linking densities through electrostatic interactions between QCS^+^–PB^−^; such combined effects result in a decrease in dry-state pore size due to physical filling. In contrast, DPB diminishes the integrity of the cross-linking network due to charge neutralization via PDA. Therefore, their contribution to pore size reduction is primarily attributed to particle filling.

### 2.2. Physical Property of DPB-ODQ Hydrogels

#### 2.2.1. Swelling Performance

The swelling process of ODQ composite hydrogels in PBS can be categorized into two distinct stages: During the initial stage (0–1 h), rapid swelling occurred, with mass increasing rapidly beyond 400% of its initial dry weight within one hour. Subsequently, during the second stage (1–12 h), a significant decrease in the swelling ratio was observed until equilibrium was reached around 12 h, yielding values of 973%, 868%, and 730% (Figure 3e). This alteration may be attributed to the introduction of nanofillers: On the one hand, NPs increase the cross-linking density within the hydrogel network; on the other hand, they modify its micro-porous structure, thereby influencing the water absorption properties within this system. Specifically, DPB exhibits both enhanced chemical cross-linking effects and hydrophobic surface characteristics that significantly inhibit swelling behavior, while unmodified PB provides only limited suppression of swelling through physical filling mechanisms. Clearly, the ODQ series maintains long-term structural integrity and fulfills the requirements for wound dressing due to its superior swelling behavior.

#### 2.2.2. Gelation Time

In the ODQ hydrogel system, the incorporation of both PB and DPB markedly reduced gelation time (GT) (*p* < 0.001; Figure 3f). The average GT for PB-ODQ was found to be 62 s, whereas that for DPB-ODQ was reduced to 45 s (its transition from solution to gel depicted in Figure 3g), which can be attributed to coordination interactions between the surface Fe^3+^ of PB and –NH_2_ groups present in CS, thereby providing additional cross-linking points. NPs act as physical cross-linking centers that facilitate network formation; however, particle aggregation may impede enhancements in cross-linking efficiency. The PDA modification of PB improves NP dispersion by exposing a greater number of active sites.

#### 2.2.3. Degradation Performance

Biodegradability is a critical criterion for the evaluation of hydrogels. Quantitative analyses revealed differences in mass retention rates (MRRs) over a three-day period (Figure 3h). Specifically, on the 3rd day, the MRR for ODQ was 55.36%, whereas it was improved to 66.55% for PB-ODQ and 73.20% for DPB-ODQ. PB and DPB delay the degradation process by enhancing the cross-linking network’s density. Notably, after an 11-day period, despite the final MRR dropping below 25%, differences remained: ODQ exhibited the fastest degradation rate, followed by PB-ODQ, while DPB-ODQ demonstrated superior stability. This discrepancy may arise from the dual role of the PDA coating: on the one hand, PDA prevents NP aggregation, ensuring uniform dispersion; on the other hand, it further enhances network stability by introducing additional cross-linking sites. We also conducted a preliminary stability experiment by storing DPB-ODQ at 4 °C for seven days. The results indicated that it maintained good physical integrity without significant dehydration or morphological changes during this period (Figure S3). Furthermore, there were no notable changes in its PT capacity between day one and day seven (Figure S4). In addition, DPB demonstrated a slow and continuous release profile from DPB-ODQ, exhibiting a cumulative release rate of less than 16% by day nine (Figure S5). However, real-time stability monitoring and more comprehensive assessments regarding chemical stability, such as evaluations concerning characteristic functional group stability and oxidation resistance, require further enhancement in future research endeavors.

#### 2.2.4. Injectable, Self-Healing, and Adhesive Properties

**Figure 4:**
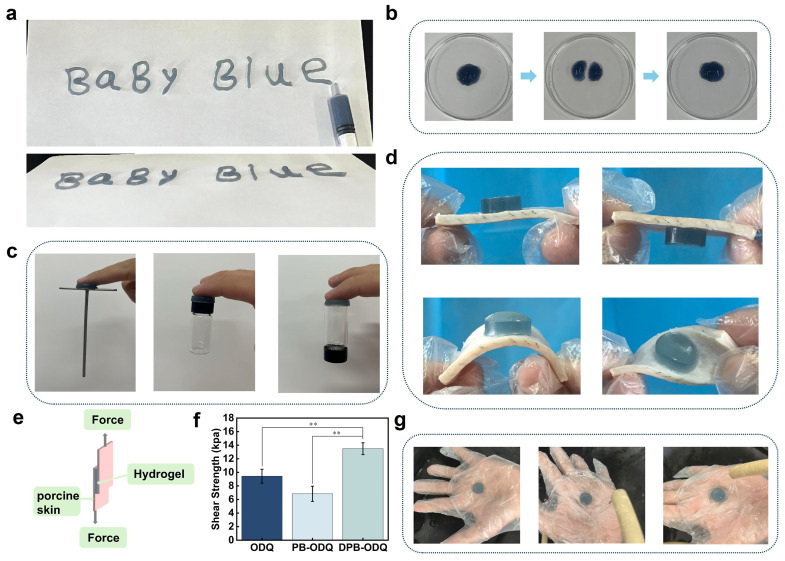
Evaluation of injectable, self-healing, adhesive and fluidity properties in the hydrogels: (**a**) Injectability assessment. (**b**) Photographic evidence demonstrating self-healing capability. (**c**) Images depicting adhesion capabilities to plastic, glass, and steel surfaces. (**d**) Adhesion performance on pig skin. (**e**) Schematic representation outlining the adhesion shear test. (**f**) Adhesive strength between the hydrogels and pig skin (** *p* < 0.01, *n* = 3). (**g**) Resistance evaluation against water flow impact.

When applied to wound sites, injectable hydrogels can precisely adapt to wound size, thereby facilitating the coverage of irregularly shaped injuries. DPB-ODQ could be extracted via syringe into a form resembling “Baby Blue” (Figure 4a). Notably, when severed into two pieces, it seamlessly reattached after merely 2 h of close contact at 25 °C without any external intervention (Figure 4b). Dressings with enhanced tissue adhesion contribute to the formation of a robust physical barrier that effectively prevents external damage while fostering an optimal healing environment. It was evident that DPB-ODQ demonstrated excellent adhesion to plastic, glass, and metal surfaces (Figure 4c), forming a strong bond with pig skin while maintaining structural integrity even under bending and twisting conditions (Figure 4d). A lap shear test was performed to further assess adhesive strength (AHS; Figure 4e). The AHS of ODQ was measured at 8.7 kPa; however, upon incorporating NPs, the AHS of PB-ODQ decreased to 6.8 kPa. In contrast, DPB-ODQ demonstrated a significant increase to 13.5 kPa (*p* < 0.01; Figure 4f). Furthermore, when subjected to dynamic forces from water flow, DPB-ODQ exhibited remarkable stability, with no signs of deformation or detachment observed (Figure 4g), underscoring its suitability for applications involving frequent movement while minimizing fixation and reducing infection risks.

#### 2.2.5. Rheological Properties

The curves of the storage (G′) and loss (G″) moduli indicated that the elastic modulus of DPB-ODQ was the highest among the samples tested (Figure S6). Its observed increase in G’ may be attributed to the role of rigid DPB as an effective nanofiller, which can withstand and distribute applied stress, thereby exhibiting a macroscopically higher modulus. Furthermore, comparisons with PB-ODQ and ODQ confirmed that the incorporation of NPs is not only essential but also that their doping modification further optimizes interfacial interactions, mitigates particle agglomeration, and achieves a more pronounced enhancement effect. This results in an increased storage modulus and improved stability. The structural recovery scan clearly demonstrated that G’ and G″ intersected at around 1000% strain (Figure S7). Subsequent samples revealed that at lower strains (1%), G’ exceeded G″, indicating that the hydrogel operates within its elastic region; conversely, when subjected to high dynamics where G’ fell below G″, it signified network collapse. Within 100 s following stress application, G’ returned to its original pre-fracture state value, showcasing significant recovery capabilities after three cycles. Throughout this process, both G’ and G″ nearly reverted to their initial values.

#### 2.2.6. Photothermal Property

**Figure 5:**
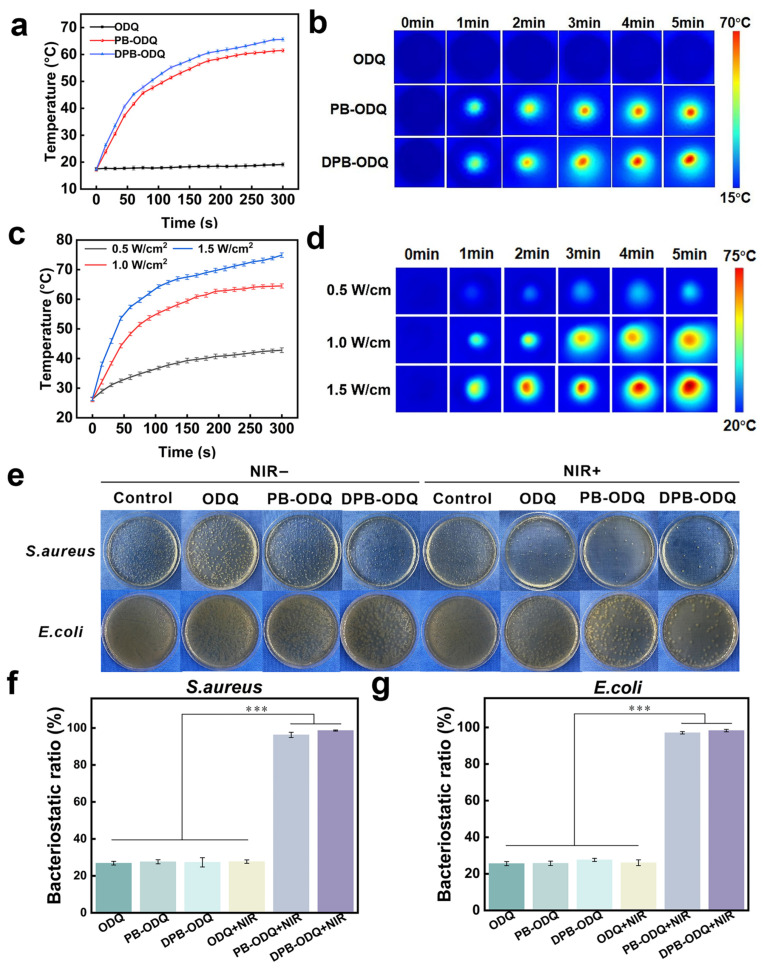
PT properties and antibacterial activity evaluation of hydrogels: (**a**) Temperature distribution across three different hydrogels under 1.0 W/cm^2^ laser irradiation (*n* = 3). (**b**) Thermal imaging corresponding to (**a**). (**c**) Temperature profiles observed in the DPB-ODQ hydrogel under varying power laser irradiation (*n* = 3). (**d**) Thermal imaging corresponding to (**c**). (**e**) Photographs depicting colony growth on Petri dishes following various treatments. (**f**) Antibacterial activity assessment against *S. aureus* (*** *p* < 0.001, *n* = 3). (**g**) Antibacterial activity assessment against *E. coli* (*** *p* < 0.001, *n* = 3).

PT performance tests indicated that both PB-ODQ and DPB-ODQ exhibited effective PT conversion capabilities under NIR illumination at 1 W/cm^2^ (Figure 5a). Based on ITI results, after 5 min of irradiation, the temperatures of both composites increased by more than 35 °C; The η of DPB was calculated as 30.41% [27] (Figure S8). In comparison, LCPN hydrogels demonstrated a maximum temperature increase of less than 30 °C [28], highlighting the superior PT capacity of DPB-ODQ. The composition and structure of PB NPs facilitate specific electronic transitions or vibrational responses upon exposure to NIR light, effectively absorbing energy. Upon the absorption of light energy, electrons in PB are excited to elevated energy levels; these excited-state electrons rapidly convert absorbed light energy into thermal energy through non-radiative transitions. This thermal energy is then transferred to the surrounding environment via heat conduction and diffusion, ultimately resulting in a PT response [29]. In contrast, negligible temperature changes were observed in ODQ (Figure 5b). Notably, the temperature rise in DPB-ODQ was slightly greater than that observed in PB-ODQ, which may be attributed to the enhanced η provided by the PDA coating layer. The temperature change in DPB-ODQ exhibited a clear positive correlation with laser power (Figure 5c). The corresponding ITI results intuitively illustrated this power-dependent temperature increase process (Figure 5d).

### 2.3. Biological Evaluation of DPB-ODQ Hydrogels

#### 2.3.1. Antibacterial Properties

The WH process is significantly influenced by bacterial infections, which not only delay tissue repair but can also exacerbate patient pain and result in systemic infections, ultimately increasing medical costs. Although QCS possesses certain antibacterial properties due to the –NH_2_ groups within its molecular structure, this singular antibacterial mechanism is insufficient for meeting the stringent clinical requirements in WD. In recent years, PT antibacterial therapy has emerged as a prominent area of research within infection treatment due to its rapid action, good selectivity, and low propensity for developing resistance. Antibacterial performance tests indicated no significant difference in bactericidal rates (BRs) for ODQ with or without NIR irradiation at 0.5 W/cm^2^ over a duration of 5 min (*p* > 0.05), suggesting that NIR irradiation alone does not exhibit bactericidal effects (Figure 5e–g). BR values for PB-ODQ and DPB-ODQ were below 30%, falling short of meeting WD requirements. Fortunately, under NIR exposure, both PB-ODQ and DPB-ODQ demonstrated a significant reduction in bacterial viability, with BR exceeding 96% following 5 min of NIR radiation (*p* < 0.001). The biofilm inhibition experiments revealed that the combination of DPB-ODQ with laser treatment significantly inhibited biofilm formation (*p* < 0.05; Figures S9 and S10). On the one hand, QCS interacts electrostatically with bacterial cell membranes through its cationic groups, compromising membrane integrity and increasing permeability. On the other hand, localized hyperthermia generated by DPB under NIR irradiation induces bacterial protein denaturation and DNA damage [29]. Importantly, initial membrane disruptions caused by QCS create favorable conditions for the deeper penetration of the PT effect into bacterial cells while further exacerbating disorganization within the membrane structure due to thermal effects. This synergistic antibacterial strategy effectively overcomes limitations associated with single-mechanism approaches, thereby achieving enhanced bacterial eradication.

#### 2.3.2. Cytocompatibility

**Figure 6:**
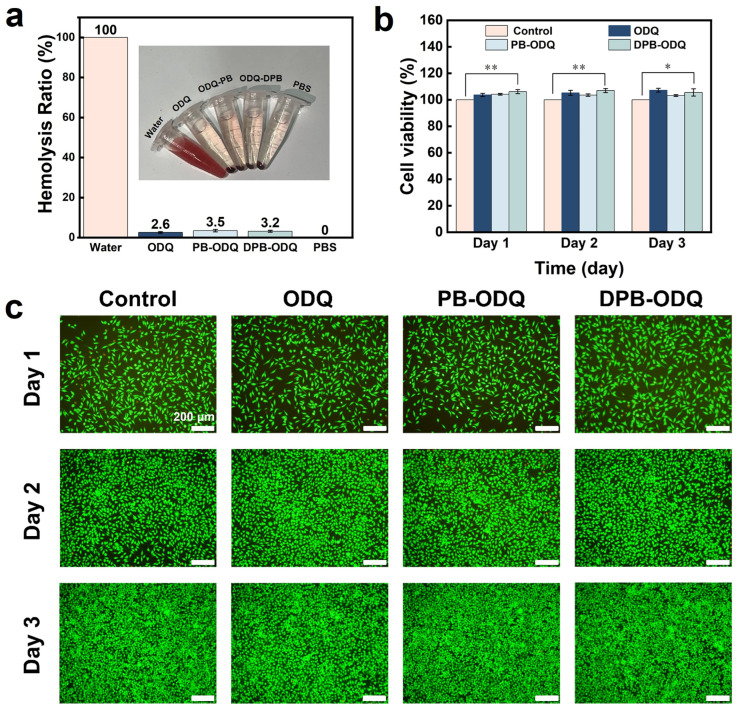
Cytocompatibility assessment of hydrogels: (**a**) Hemolysis observed in erythrocytes exposed to hydrogels (*n* = 3). (**b**) Viability analysis of L929 cells co-cultured with hydrogels (* *p* < 0.05, ** *p* < 0.01, *n* = 3). (**c**) Live/dead staining images depicting L929 cells co-cultured with hydrogels for durations of 1, 2 and 3 days, respectively.

Given that the initial stages of WH are often accompanied by bleeding, an ideal hemostatic material must exhibit excellent cytocompatibility. We conducted an examination by co-culturing ODQ composite hydrogels with RBC suspensions, which revealed negligible color differences compared to PBS, with all samples demonstrating hemolysis rates below 4%; this confirmed their good hemocompatibility (Figure 6a), which is attributable to the inherent biosafety characteristics of their constituent components.

The CCK-8 assay data indicated that the metabolic activity of L929 cells co-cultured with hydrogels consistently increased over a 3-day culture period, with cell proliferation rates exceeding 100% (Figure 6b). This not only confirms the absence of cytotoxicity but also suggests their potential role in promoting cell growth. Furthermore, live/dead staining results supported the same conclusion, as most cells within the culture system exhibited green fluorescence indicative of viable cells, while only sporadic red fluorescence marking dead cells was observed (Figure 6c). Notably, cell counts demonstrated a stable growth trend throughout the culture period, indicating that L929 cells maintained normal proliferative activity in their presence. Therefore, these meticulously selected raw materials provide reliable safety assurance for clinical applications involving DPB-ODQ.

#### 2.3.3. In Vivo Wound Healing Assessment

**Figure 7:**
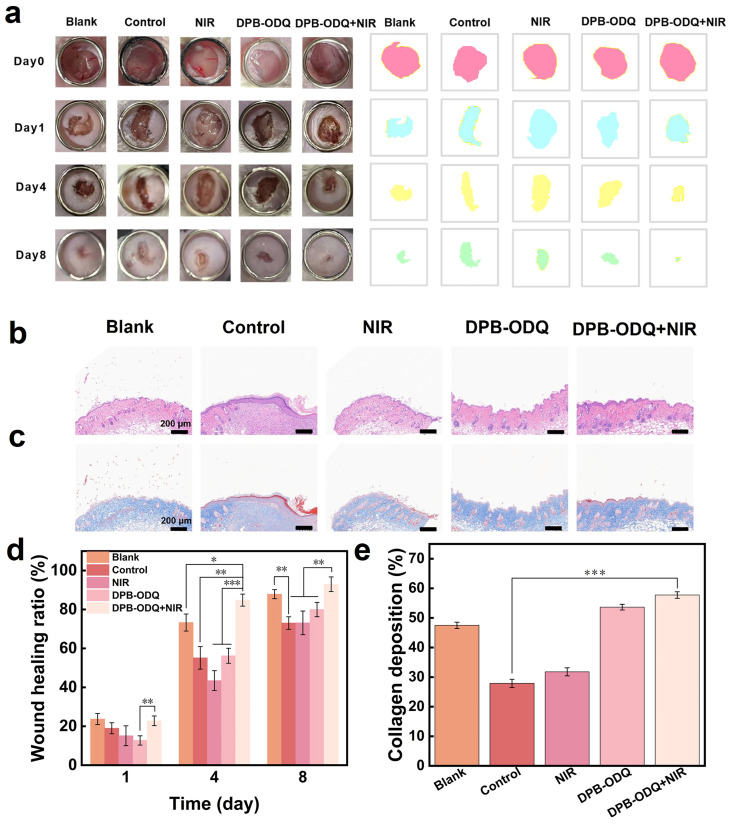
In vivo evaluation of healing in infected wounds: (**a**) Representative photographs of wounds along with schematic diagrams illustrating the wound area on days 0 (red), 1 (blue), 4 (yellow), and 8 (green). (**b**) Representative micrographs of H&E staining from five groups on day 8. (**c**) Representative micrographs of Masson staining from five groups on day 8. (**d**) WHR across five different treatment groups at days 0, 1, 4, and 8 (* *p* < 0.05, ** *p* < 0.01, *** *p* < 0.001, *n* = 3). (**e**) Collagen deposition rates among five treatment groups (*** *p* < 0.001, *n* = 3).

To investigate the efficacy of DPB-ODQ in promoting WH in vivo, we employed a mouse model with wounds infected by *Staphylococcus aureus*. Notably, the wound areas across all groups exhibited a progressive reduction over time (Figure 7a). Furthermore, we calculated the wound healing rate (WHR) at three distinct time points by quantifying the wound areas (Figure 7d). Specifically, on day 8, compared to the untreated control group (87.88%), WHR in infected individuals within the control cohort was significantly lower at 73.04% (*p* < 0.01), confirming that bacterial infection impedes tissue regeneration efforts. Although some healing-promoting effects were observed with DPB-ODQ alone (79.98%), its overall effectiveness did not reach optimal levels due to QCS’s inherent limited antibacterial activity. However, when combining DPB-ODQ with NIR light exposure, we obtained remarkable therapeutic outcomes; notably, we attained a WHR of 92.82%, which surpassed both NIR treatment alone (73.13%) and DPB-ODQ monotherapy (*p* < 0.01). This enhanced performance may be attributed to the potent bactericidal action induced by NIR light within this composite system. Figure 7b shows the HE staining images of skin tissues on day 8. In comparison to the blank group, both control and NIR-treated groups displayed increased infiltration of inflammatory cells, indicating that the lack of intervention during bacterial infection prolongs healing by maintaining an inflammatory state at the wound site. Importantly, treatment with DPB-ODQ combined with NIR revealed tumor-like follicles and vascular tissue formation, suggesting that this combination can effectively enhance the healing process for infected wounds. DPB-ODQ combined with NIR irradiation also resulted in a markedly higher rate of angiogenesis compared to the control group (*p* < 0.001; Figure 7b and Figure S11), and this rate was around 2.4-fold greater. Furthermore, collagen deposition is closely associated with tissue remodeling. Masson staining revealed that, among all treatment groups, the DPB-ODQ+NIR group exhibited the most pronounced collagen deposition (Figure 7c,e), which was about 2.1 times higher than that observed in the control group. In contrast, collagen deposition in the LCPN+NIR group was increased by about 1.3-fold relative to the control [28]. These findings underscore the robust WH capabilities of DPB-ODQ, which are attributed to the synergistic effects of the QCS and PDA coatings. QCS possesses inherent antibacterial properties and has been shown to promote fibroblast proliferation and collagen synthesis [30,31]. PDA is rich in catechol functional groups, enabling it to function effectively as an electron donor for the direct reduction and neutralization of ROS. Moreover, studies have suggested that PDA exhibits intrinsic enzyme-mimetic activities akin to those of peroxidase and superoxide dismutase, allowing it to catalytically decompose ROS [17]. Consequently, through its chemical reductive capacity and potential enzymatic mimicry, the PDA coating establishes an effective antioxidant barrier at the wound site. To assess systemic biocompatibility, hematoxylin and eosin (H&E) staining was performed on major organs of mice following treatment. No evident inflammatory cell infiltration or histopathological abnormalities were observed in the DPB-ODQ+NIR group when compared with healthy tissues from control animals (Figure S12). These results indicated that DPB-ODQ demonstrates minimal biological toxicity toward normal tissues in vivo.

Although this study has demonstrated the significant short-term efficacy of the DPB-ODQ hydrogel in treating infected acute wounds, we acknowledge several limitations inherent in the current animal experimental design. First, the relatively short evaluation period prevents a comprehensive assessment of long-term effects on tissue remodeling and scar formation. Second, the absence of a control group receiving an established standard of care, such as silver-based dressings, limits our ability to ascertain its relative therapeutic superiority. Furthermore, this study employed an acute infected wound model that may not fully capture the complex pathophysiological conditions encountered in chronic wounds. Given the multifunctional properties of DPB-ODQ, it holds promising potential for application in more challenging healing environments, including chronic wound settings such as diabetic foot ulcers. Addressing these limitations through future studies will be a key focus of our ongoing research efforts.

## 3. Conclusions

In this study, a multifunctional Prussian-blue-based nanocomposite hydrogel DPB-ODQ was fabricated via a Schiff-base reaction between QCS and ODex doped with DPB NPs. The obtained DPB-ODQ hydrogel exhibited multiple outstanding functional properties: rapid gelation, injectability, self-healing ability, and good adhesion. Due to the existing DPB NPs, hydrogels exhibited exceptional PT performance, enabling rapid heating under 808 nm laser irradiation and achieving sterilization efficiency exceeding 96% against *S. aureus* and *Escherichia coli*. In addition, the hydrogel significantly promoted fibroblast proliferation. Finally, the mouse model experiments confirmed that it also possessed good tissue compatibility and effectively accelerated the WH process, emphasizing that DPB-ODQ hydrogels can serve as a novel dressing for treating infected wounds.

## 4. Materials and Methods

### 4.1. Materials

QCS (degree of substitution: 40%) and potassium ferricyanide (K_3_[Fe(CN)_6_]·3H_2_O) were procured from Shanghai Macklin Biomedical Co., Ltd. (Shanghai, China), while sodium periodate (NaIO_4_) and ethylene glycol were obtained from Tianjin Yongda Chemical Reagent Co., Ltd. (Tianjin, China). Polyvinylpyrrolidone (PVP) was sourced from Tianjin Guangfu Technology Development Co., Ltd. (Tianjin, China). DA hydrochloride and Dex were obtained from Shanghai Aladdin Biochemical Technology Co., Ltd. (Shanghai, China). Tris-HCl buffer (50 mM, pH = 8.5) and PBS (10 mM, pH = 7.4) were purchased from Shenggong Biotechnology (Shanghai) Co., Ltd. (Shanghai, China). Two pathogenic bacterial strains—including *S. aureus* Z-403S and *E. coli* Z-402E—were isolated from clinical specimens in our laboratory. They were identified through morphological observation coupled with 16S rRNA gene sequence analysis. Female Kunming mice aged between six to seven weeks were obtained from (China) Henan Kebisi Biotechnology Co., Ltd. (Zhengzhou, China).

### 4.2. Synthesis of Prussian Blue Nanoparticles

At 25 °C, 600 mg of K_3_[Fe(CN)_6_]·3H_2_O was dissolved in a solution containing 6 g of PVP and 80 mL of 0.01 M hydrochloric acid (HCl). Then, the mixture was stirred magnetically for one hour. The resulting mixture was transferred to a water bath at 80 °C and subjected to heat treatment for 20 h. Following the reaction, the product was centrifuged at 3500 rpm for 5 min. The precipitate was alternately washed with deionized water (DI) and anhydrous ethanol (AE) three times each. Finally, the obtained solid was dried overnight in a vacuum oven at 60 °C, yielding deep blue PB NPs.

### 4.3. Synthesis of PB@PDA Nanoparticles

PB NPs (18 mg) were dispersed in 60 mL of Tris-HCl buffer containing DA (18 mg). This mixture underwent ultrasonic treatment for 10 min to ensure proper dispersion. Next, it was continuously stirred at 25 °C for 10 h to facilitate the coating process of PDA onto PB NPs. The resultant DPB precipitate was collected via centrifugation and subsequently washed alternately with DI and AE three times each before being dried overnight in a vacuum oven at 60 °C.

### 4.4. Synthesis of ODex and Determination of Its Oxidation Degree

Dex (4 g) and NaIO_4_ (3.6 g) were dissolved in 400 mL DI under dark conditions while stirring at 25 °C for 24 h to facilitate the oxidation reaction. After this period, 2 mL of ethylene glycol was introduced to terminate the reaction process. The resulting solution underwent purification using dialysis bags with a molecular weight cut-off range between 8000 and 14,000 Da over 3 days; ultimately, the dialysate was freeze-dried to yield white spongy ODex [22].

The freeze-dried ODex (0.1 g) was weighed and transferred to a 250 mL conical flask. A 0.5 M hydroxylamine hydrochloride (HAH) solution (20 mL) was added, ensuring thorough mixing to fully dissolve or disperse the sample. Two to three drops of bromophenol blue indicator were introduced; at this stage, the solution should appear yellow, indicating an acidic environment. The flask was covered with a surface dish and placed in a water bath maintained at 40 °C for a duration of 2 h to ensure the complete reaction of –CHO groups. Upon completion of the reaction, the conical flask was allowed to cool to 25 °C. A standardized NaOH solution (0.1 M) was titrated until a color change from yellow to blue-purple was observed, and the volume of NaOH consumed was recorded as V_sample_. Simultaneously, a blank experiment was conducted by omitting ODex while maintaining identical volumes for the HAH solution and indicator; all previous steps were repeated, and the volume of NaOH consumed was documented as V_blank_. The oxidation degree of ODex was calculated using Equation (1):

oxidation degree (%) = [C_NaOH_ × (V_blank_ − V_sample_) × M_glucose_]/(m_sample_ × 1000) × 100 (1)

where C_NaOH_ represents the concentration of the NaOH standard solution (mol/L), M_glucose_ denotes the molar mass per glucose unit (162 g/mol), m_sample_ indicates the mass of ODex in g, and 1000 serves as a conversion factor from L to mL.

### 4.5. Preparation of DPB-Incorporated Polysaccharide Hydrogels

To prepare the hydrogels incorporating DPB, 1 mL of QCS solution (3.0% *w*/*v*: 0.3 g of QCS powder dissolved in 10 mL of DI using a magnetic stirrer at 350 rpm for 20 min) containing PB NPs was mixed with another 1 mL of QCS solution containing 0.5 mg/mL DPB, along with 1 mL of ODex solution (3.0% *w*/*v*: 0.3 g of white spongy ODex dissolved in 10 mL of DI under the same conditions as the 3.0% QCS solution preparation): (1) ODQ group (control): QCS solution (1 mL; without PB NPs) + ODex solution (1 mL); (2) PB-ODQ group: QCS solution (1 mL; 0.5 mg/mL of PB NPs) + ODex solution (1 mL); (3) DPB-ODQ group: QCS solution (1 mL; 0.5 mg/mL of DPB) + ODex solution (1 mL). After mixing within each group, they underwent cross-linking via dynamic Schiff-base reactions and were allowed to stand for 10 min, resulting in ODQ, PB-ODQ, and DPB-ODQ hydrogels, respectively.

### 4.6. Structure Characterization of Materials and Hydrogels

#### 4.6.1. Transmission Electron Microscope

A small amount of dry PB and DPB was ground into powder form. They were then ultrasonically dispersed using AE before being deposited onto carbon-supported copper mesh. Following drying at 25 °C, morphological characterization was conducted using an Thermo Fisher Scientific FEI Tecnai 12 transmission electron microscope (TEM: (Waltham, MA, USA) [32].

#### 4.6.2. Nanoparticle Size Test

The dry DPB was ultrasonically dispersed in AE for 10 min before being tested with a nanoparticle size analyzer (Malvern Zetasizer Nano ZS90 from Malvern Instruments Ltd., Malvern, UK). The sample test range was set between 80 and 700 nm.

#### 4.6.3. Fourier Transform Infrared Spectrometer

To verify the successful synthesis of DPB and ODex, both PB (DPB) and Dex (ODex) were analyzed using the KBr pellet method. Subsequently, a Fourier transform infrared spectrometer (FTIR: Thermo Fisher Scientific Nicolet iS20 from Thermo Fisher Scientific Inc., Waltham, MA, USA) was employed within a scanning range of 4000–400 cm^−1^.

#### 4.6.4. Ultraviolet–Visible Absorption Spectroscopy

The dry PB and DPB were diluted in DI. Using DI as a blank reference, full-band scanning was performed within the wavelength range of 300–850 nm utilizing a UV-1800 ultraviolet–visible spectrophotometer from Shimadzu Corporation (Kyoto, Japan). Sample solutions containing varying concentrations of PB and DPB were sequentially injected into the cell, with their absorption spectra recorded. Finally, we determined the characteristic absorption peak at 808 nm and noted its optical density (OD).

#### 4.6.5. Scanning Electron Microscope

Fully cross-linked ODQ, PB-ODQ, and DPB-ODQ hydrogels were subjected to freeze-drying for 48 h to obtain a complete porous network structure. Cross-sectional samples were prepared for fixation on the sample stage, followed by gold sputtering treatment. Subsequently, microscopic morphology was examined using a German ZEISS Sigma 360 scanning electron microscope (SEM) from Carl Zeiss AG (Oberkochen, Germany) [33].

### 4.7. Physical Characterization of Hydrogels

#### 4.7.1. Swelling Behavior

The swelling behavior was systematically evaluated at 25 °C using PBS. Each lyophilized hydrogel was weighed (W_0_), followed by complete immersion in PBS. At predetermined intervals—namely, 1, 2, 3, 6, 12, and 24 h—they were extracted and gently blotted with filter paper to absorb any excess liquid droplets while preventing deformation due to compression. The mass of swollen hydrogels was rapidly weighed and denoted as W_t_. The swelling ratio was calculated using Equation (2):

swelling ratio (%) = [(W_t_ − W_0_)/W_0_] × 100 (2)

where W_t_ signifies the swelling equilibrium wet weight at time t, and W_0_ represents the initial dry weight of the hydrogel.

#### 4.7.2. Degradation Tests

In vitro degradation studies were also conducted using PBS. Hydrogels with consistent sizes and shapes were prepared. After drying, their initial mass was recorded as W_0_; then, they were added to a container filled with sufficient PBS and incubated at 37 °C. At predetermined time points, the hydrogels were retrieved, dried again, and weighed (W_t_). The degradation rate (also called MRR) was calculated using Equations (3) and (4):

degradation rate (%) = [(W_0_ − W_t_)/W_0_] × 100 (3)

MRR (%) = 100 − degradation rate (%)(4)

where W_0_ denotes the initial dry weight, and W_t_ represents the dry weight after time t has elapsed.

To directly evaluate the release behavior and structural stability of DPB from the DPB-ODQ hydrogel, we utilized a monitoring method based on its characteristic absorbance. In total, 200 mg of DPB-ODQ was dispersed in 50 mL of PBS buffer through vortex oscillation. Following centrifugation (5500 rpm, 10 min), the supernatant was collected, and its OD_700_ was measured. This value is denoted as OD_total_ and serves as a reference for the total amount of DPB contained within the hydrogel. An equal mass of hydrogel was then placed into 50 mL of PBS release medium and continuously shaken at 37 °C with an agitation speed of 100 rpm. OD_t_ denotes predetermined time points (such as days 1, 3, 5, 7, and 9); samples measuring 1 mL were withdrawn from the release medium and immediately subjected to centrifugation (5500 rpm, 10 min) to remove any unreleased NPs and hydrogel fragments. Subsequently, the OD_t_ of the supernatant at 700 nm was determined; this signal directly reflects structurally intact DPB released into the medium. The cumulative release rate of DPB was calculated using Equation (5):

cumulative release rate (%) = (OD_t_/OD_total_) × 100 (5)

#### 4.7.3. Self-Healing Tests

The complete DPB-ODQ hydrogel was cut into two independent fragments. Both cut surfaces were closely aligned and maintained in contact at 25 °C for 2 h to facilitate reorganization and repair within the internal dynamic cross-linking network. After this step, macro-morphological images capturing its self-healing interface were documented.

#### 4.7.4. Adhesion Tests

An adhesion test was first conducted on plastic, glass, metal, and pig skin. Subsequently, the AHS was quantitatively evaluated using lap shear strength tests. Fresh pig skin tissue was precisely trimmed to remove excess fat and cut into rectangular samples measuring 1 cm × 2 cm. Hydrogels (200 μL) were evenly applied to the bonding area (10 × 10 mm^2^) between two pig skins. To promote interfacial bonding and facilitate curing, a constant pressure of 50 g was exerted on the bonding surface and maintained at 25 °C for 0.5 h. Following the curing process, they were placed in a tensile testing machine with JiTai-W500 N from Jitai Technology Co., Ltd. (Keelung, China) and subjected to tensile loading at a constant displacement rate of 10 mm/min until the complete separation of the bonding interface occurred, thereby determining AHS.

#### 4.7.5. Rheological Analysis

The freeze-dried ODQ, PB-ODQ, and DPB-ODQ hydrogels were placed on the stage of a rotational rheometer with Haake Mars 60 from Thermo Electron (Karlsruhe) GmbH (Karlsruhe, Germany) at 25 °C. A dynamic oscillation frequency sweep was conducted over a range from 1 Hz to 100 Hz while maintaining a fixed strain of 1%. Rheological data were subsequently recorded and graphically analyzed.

The strain sweep tests involved setting parameters—such as strain ranging from 1% to 1000%—while keeping the temperature constant at 25 °C, and an angular frequency of 1 rad/s was used. The sweep progressed from low to high strain values. Force application was halted each time the hydrogel fractured under pressure until self-healing occurred. The strain was then incrementally increased again until failure reoccurred, allowing for multiple test cycles. The rheological properties were evaluated based on G′ and G″.

#### 4.7.6. Photothermal Tests

Equal masses (200 mg) of ODQ, PB-ODQ, and DPB-ODQ were placed into wells within a 48-well plate and irradiated with an 808 nm NIR laser system for 5 min at 1 W/cm^2^. The dynamic changes in surface temperature were monitored in real time using an infrared thermal imaging camera (ITI) with FLIR E60 from FLIR Systems, Inc. (Wilsonville, OR, USA). Gradient power irradiation was applied to DPB-ODQ for 5 min, during which the temperature increase in the sample was recorded. According to Ren’s report [27], the photothermal conversion efficiency (η) is calculated using Equations (6)–(9): First, τ_s_ should be calculated using Equations (7) and (8), followed by calculating *h*A with Equation (9); finally, these values can be substituted into Equation (6) to obtain η:

η = [*h*A(ΔT_max_ − ΔT_max,H2O_)]/[I (1 − 10^−Aλ^)] (6)

In this equation, *h* represents the coefficient of heat transfer, A denotes the surface area, ΔT_max_ refers to the DPB solution in NIR light irradiation under the steady state’s highest temperature change, and ΔT_max,H2O_ indicates the temperature change in water under identical conditions. I signifies the NIR laser’s power density, while A_λ_ denotes the OD_808_ of DPB:

θ = [(T_surr_ − T)/(T_surr_ − T_max_)] (7)

τ_s_ = t/[−ln(θ)] (8)

where t represents the real-time cooling duration, T denotes the real-time temperature of t, T_surr_ is defined as the ambient temperature, and T_max_ corresponds to the maximum stable temperature of this solution:

*h*A = [m_H2O_C_H2O_/τ_s_] (9)

Here, m_H2O_ denotes the mass of water, and C_H2O_ represents the specific heat capacity of water; τ_s_ is identified as the time constant.

### 4.8. Antibacterial Tests

*S. aureus* and *E. coli* were selected as representative strains to evaluate the antibacterial performance of DPB-ODQ. Both cultures were adjusted to 1 × 10^6^ colony-forming units (CFU)/mL and evenly distributed (500 μL): (1) blank control group (no treatment); (2) ODQ group; (3) PB-ODQ group; (4) DPB-ODQ group; (5) blank control+NIR irradiation group; (6) ODQ+NIR irradiation group; (7) PB-ODQ+NIR irradiation group; (8) DPB-ODQ+NIR irradiation group. Among them, each test tube contained 1 mL of bacterial suspension; for the NIR irradiation groups, an 808 nm laser with a power density of 0.5 W/cm^2^ was employed. After treatment, bacterial solutions were diluted in a 100-fold gradient. The diluted solution (50 μL) was inoculated onto the surface of agar culture media using the plate coating method, followed by incubation at 37 °C for 12 h. BR values were calculated using Equation (10):

BR (%) = [(CFU_blank_ − CFU_hydrogel_)/CFU_blank_] × 100 (10)

where CFU_blank_ represents the CFU of the blank control group, and CFU_hydrogel_ indicates those from the hydrogel-treated groups.

### 4.9. Determination of Biofilm Eradication Effectiveness

The freeze-dried ODQ, PB-ODQ, and DPB-ODQ hydrogels, weighing 200 mg each, were placed in a 96-well plate. Initially, bacterial concentrations were adjusted to 10^6^ CFU/mL. Subsequently, 200 μL of the bacterial suspension was added to each well in the plate. All PT group hydrogels underwent irradiation with an 808 nm laser for 5 min (power density = 0.5 W/cm^2^), followed by co-cultivation at 37 °C for 24 h to induce biofilm formation. Upon the completion of culturing, the supernatant was carefully removed, and three gentle rinses with PBS were carried out to eliminate unattached airborne bacteria. A staining solution containing crystal violet (0.1% *w*/*v*) was then added to each experimental well and allowed to stain at 25 °C in complete darkness for 20 min. Subsequent rinsing involved three additional gentle washes with PBS to remove unbound dyes before allowing them to dry naturally. Furthermore, a solution of 95% ethanol was introduced to dissolve any adsorbed dye; the solution was then allowed to stand for 15 min, ensuring complete dissolution before measurement. OD_570_ was measured utilizing a microplate reader from Bio-Rad Laboratories, Inc. (Hercules, CA, USA).

### 4.10. Hemolysis Tests

Hemolysis tests were conducted using fresh mouse blood. Whole blood collected from mice was stored in vacuum tubes containing an EDTA-K_2_ anticoagulant. Following centrifugation at 1500 rpm for 15 min, red blood cells (RBCs) were isolated while being washed multiple times with PBS until the supernatant appeared clear, thereby obtaining 4% (*v*/*v*) of the RBC PBS suspension. Equal volumes were mixed with hydrogels, while control groups included a PBS (negative control) and a DI group (positive control). After incubating at 37 °C for 2 h, the OD_540_ of the supernatant was measured. The hemolysis rate was calculated using Equation (11):

hemolysis rate (%) = [(OD_hydrogel_ − OD_negative_)/(OD_positive_ − OD_negative_)] × 100 (11)

where OD_hydrogel_ represents the OD of hydrogel-treated groups, OD_negative_ indicates the OD of the PBS group, and OD_positive_ denotes the OD of the DI group.

### 4.11. Cytocompatibility Evaluation

In vitro biocompatibility was assessed using L929 mouse fibroblasts. Hydrogels were immersed in MEM culture medium containing 10% fetal bovine serum (*v*/*v*) and 1% penicillin-streptomycin (*v*/*v*), and the samples were extracted for 24 h at 37 °C in a 5% CO_2_ environment to prepare the extract solution, which was sterilized through a 0.22 μm filter. L929 cells were seeded into a 96-well plate at 3 × 10^3^ cells/well. After allowing for adherence over a period of 24 h, the treatment groups received their respective extract solutions (the control group utilized a complete MEM culture). Following incubation periods of 24, 48, and 72 h, the CCK-8 solution was added to each well and incubated for an additional 4 h. OD_450_ was then measured, and the cell survival rate was calculated using Equation (12):

cell survival rate (%) = [(OD_hydrogel_ − OD_blank_)/(OD_control_ − OD_blank_)] × 100 (12)

where OD_hydrogel_ refers to the OD of hydrogel-treated groups, OD_blank_ signifies the OD of the blank control group (culture medium only), and OD_control_ denotes the OD of the negative control group (cells with culture medium).

A live/dead cell double-staining experiment was also performed. L929 cells were seeded into another set of wells within a 24-well plate at 5 × 10^4^ cells/well. After culturing for 12 h, the medium was replaced with the extract solution medium while ensuring consistency in the control group, with fresh MEM provided every 24 h thereafter. Calcein-AM/propidium iodide staining occurred at three intervals post-treatment to observe morphological changes within the cells utilizing an inverted fluorescence microscope with Olympus IX53 from Olympus Corporation (Tokyo, Japan).

### 4.12. In Vivo Infected Wound Healing and Histological Analysis

To assess the in vivo antibacterial efficacy of these hydrogels along with their potential role in promoting WH processes in infected wounds, we established a skin wound model infected with *S. aureus* utilizing female Kunming mice. The mice were randomly divided into five groups: the hydrogel-free treatment group (control group), the blank group (without bacterial infection), the NIR group, the DPB-ODQ group, and the DPB-ODQ+NIR group. The fur on the back of the mice was shaved, creating a full-thickness skin defect with a diameter of 10 mm. Except for those in the blank group, all other groups were inoculated directly with 50 μL of bacterial suspension (10^6^ CFU/mL). On day one post-inoculation, mice received treatment with hydrogels. The NIR group underwent irradiation with NIR light at an intensity of 0.5 W/cm^2^ (808 nm) for 5 min. Macroscopic wound photographs were taken on days 0, 1, 4, and 8 post-operation; then, ImageJ 1.54g software was used to quantify the wound area. Healing skin tissue samples around the wound were collected on day 8 post-operation; after fixation with 4% paraformaldehyde for 48 h, they were embedded in paraffin and sectioned at a thickness of 5 μm. H&E staining was employed to assess the levels of inflammatory infiltration and re-epithelialization, while Masson’s trichrome staining facilitated the analysis of collagen deposition density under an optical microscope. WHR was calculated using Equation (13):

WHR (%) = [(1 − S_t_/S_0_)] × 100 (13)

where S_t_ represents the wound area at time t, and S_0_ denotes the initial wound area.

### 4.13. Statistical Analysis

All values are expressed as mean ± standard deviation. All quantitative experiments were independently repeated at least three times. One-way analysis of variance (ANOVA) was performed using SPSS version 22.0, followed by the SNK-q post hoc test. A *p*-value of <0.05 was defined as statistically significant (* *p* < 0.05; ** *p* < 0.01; *** *p* < 0.001).

## Data Availability

Data will be made available upon request.

## Supplementary Materials

The following supporting information can be downloaded at https://www.mdpi.com/article/10.3390/gels11110895/s1. Twelve figures (Figures S1–S12) are provided in the Supplementary Information.
