# Supplementary material for: Multifunctional Prussian-Blue-Based Nanocomposite Hydrogel for Infected Wound Regeneration
Source: Gels. 2025 Nov 8;11(11):895. doi: 10.3390/gels11110895 (PMC12652168; doi:10.3390/gels11110895)
Supplement: Supplementary file 1 [file gels-11-00895-s001.zip › gels-3927767-supplementary.pdf]

Supporting Information

Multifunctional Prussian-Blue-based Nanocomposite Hydrogel for Infected Wound Regeneration

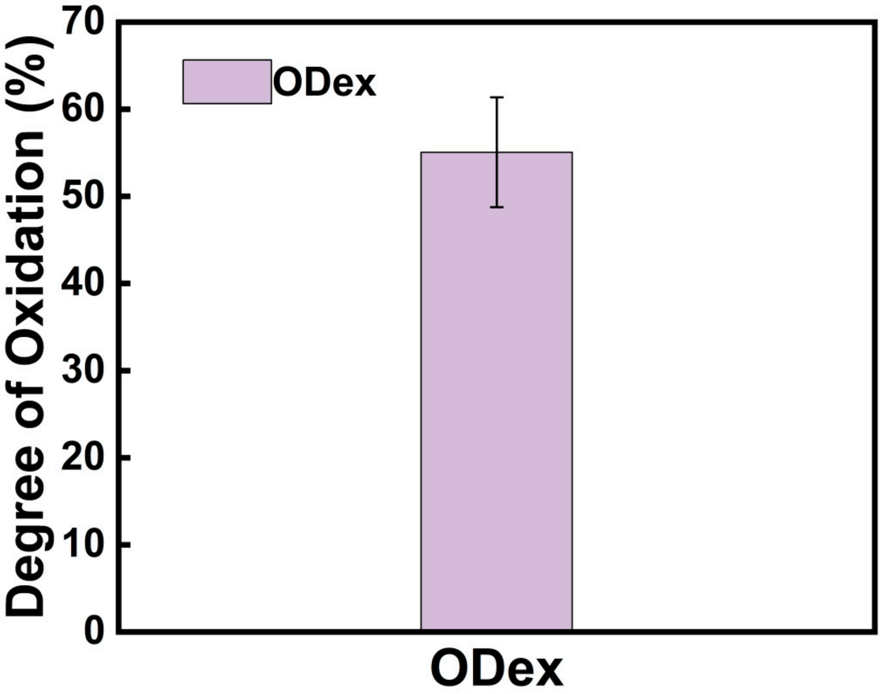

Figure S1. The oxidation degree of ODex (n = 3).

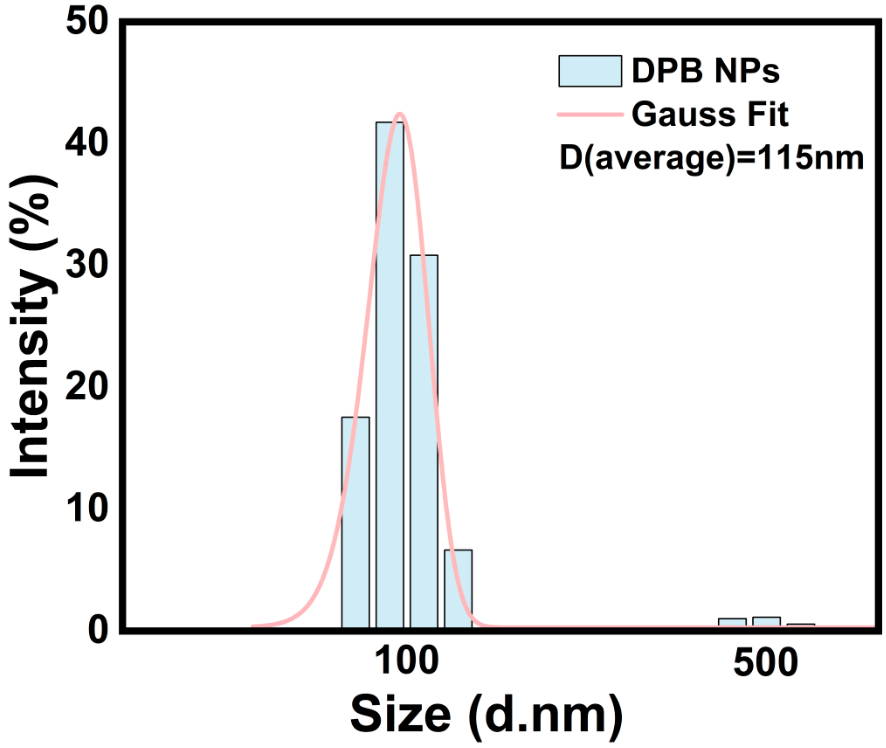

Figure S2. The size distribution of DPB NPs.

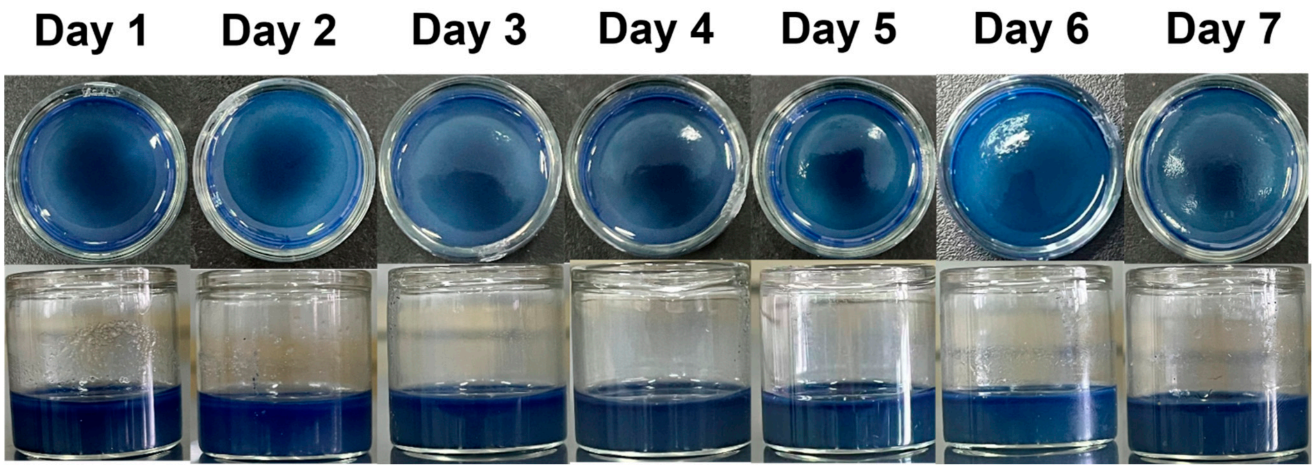

Figure S3. The morphological changes of DPB-ODQ hydrogel after being stored at 4°C for 7 days.

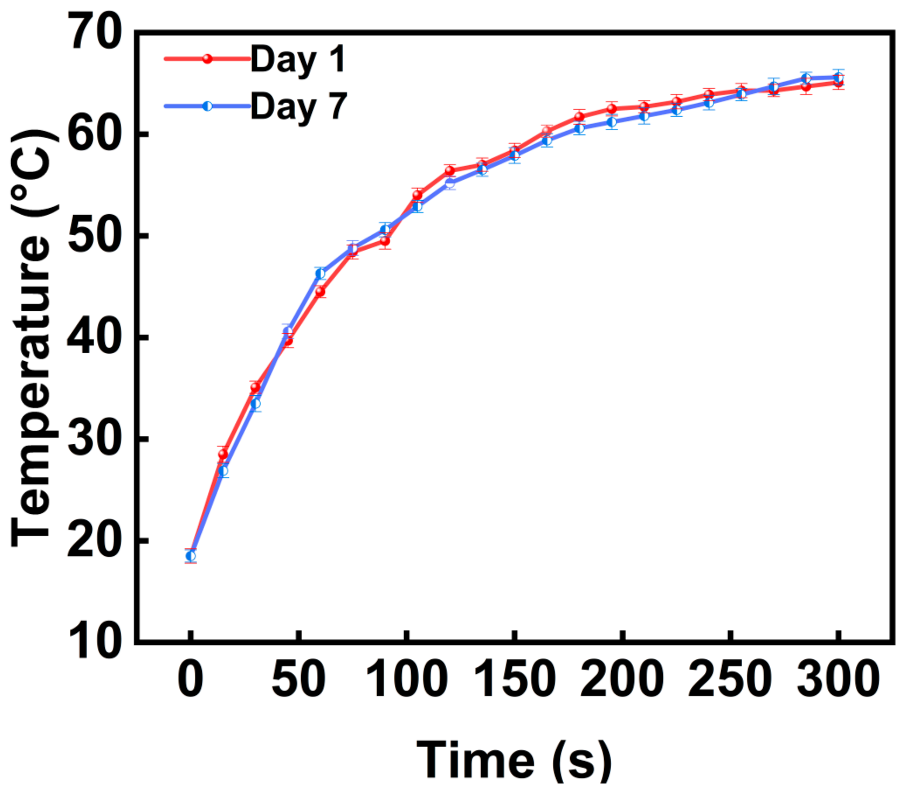

Figure S4. The photothermal changes of DPB nanoparticles on the 1st and 7th days (*n* = 3).

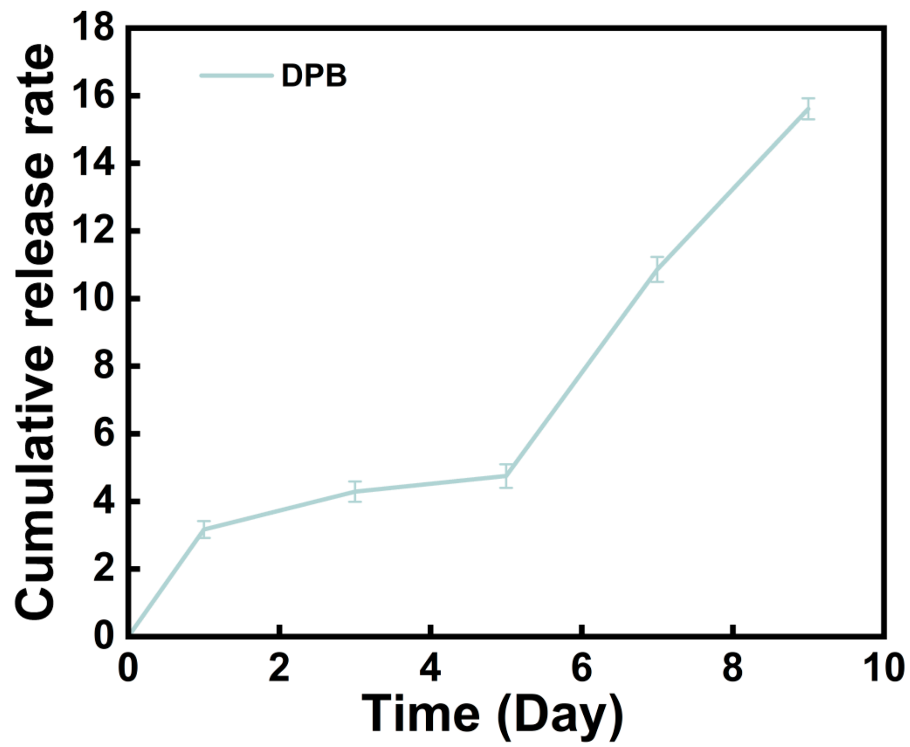

Figure S5. The cumulative release rate of DPB NPs (*n* = 3).

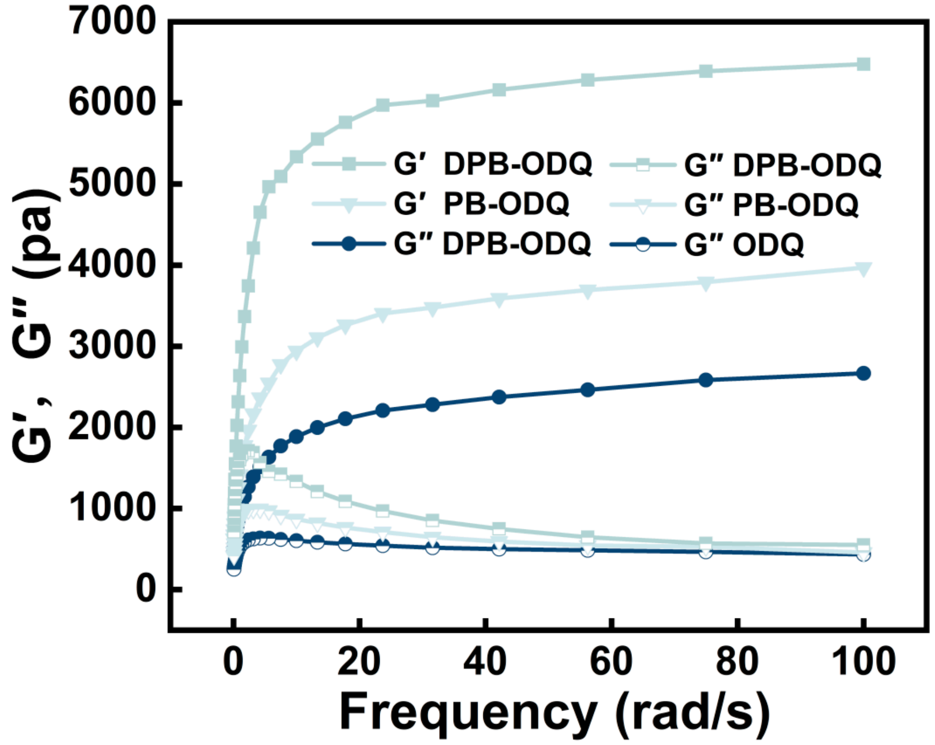

Figure S6. Rheological frequency scanning results.

.

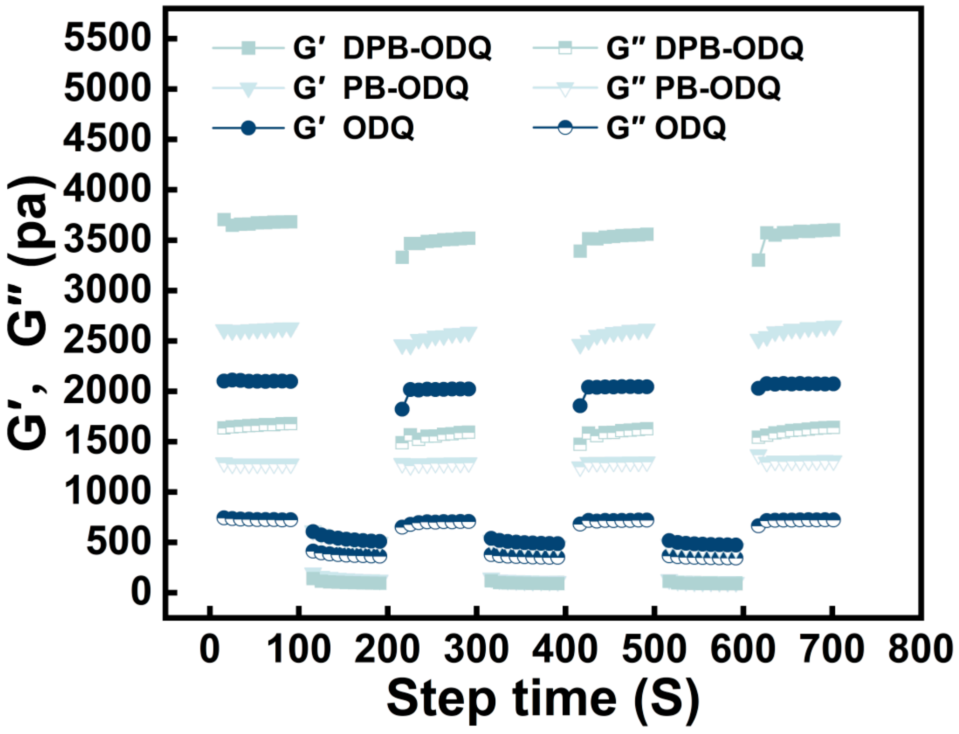

Figure S7. Rheological structure recovery scanning results.

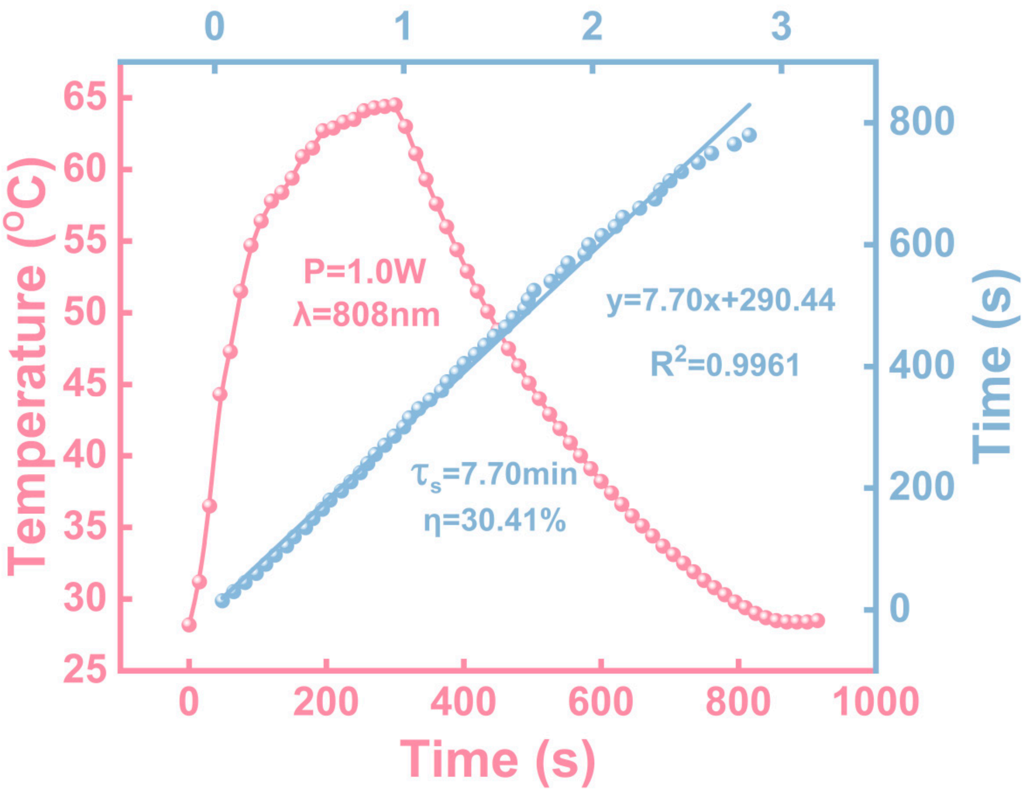

Figure S8. Photothermal curve of dispersed DPB suspension (1 mL, 1mg/mL) during on and off laser (1.0 W/cm^2).

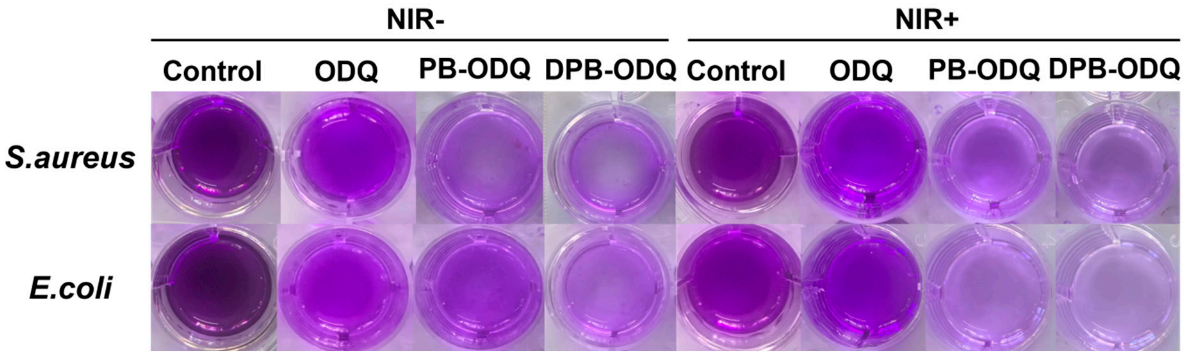

Figure S9. Biofilm eradication effectiveness of ODQ, PB-ODQ, and DPB-ODQ hydrogels.

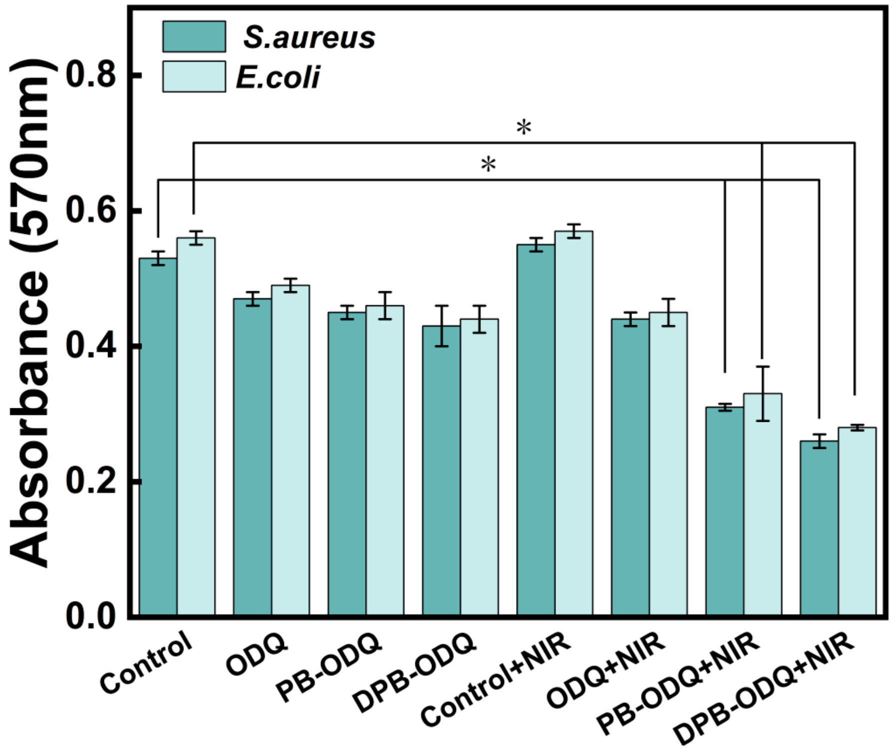

Figure S10. Quantitative analysis (OD_{570}) on biofilm eradication effectiveness of ODQ, PB-ODQ, and DPB-ODQ hydrogels (* $p < 0.05$, $n = 3$).

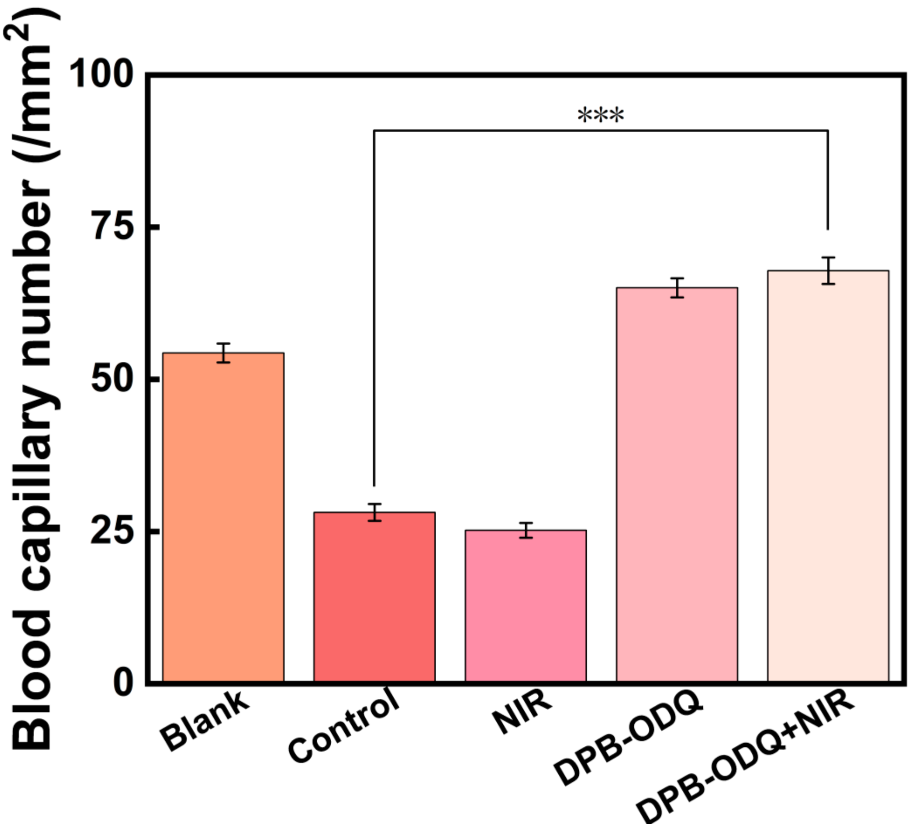

Figure S11. Quantification of capillaries (*** $p < 0.001$, $n = 3$).

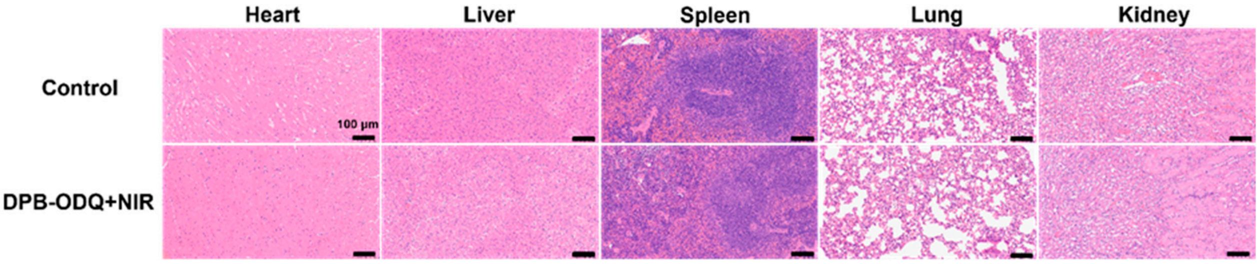

Figure S12. Histological examination of the main organs of mice by H&E staining.
